# Self-identified intervention priorities amongst women with road accident-acquired physical disabilities in South Africa

**DOI:** 10.4102/ajod.v11i0.867

**Published:** 2022-02-25

**Authors:** Laura Hartmann, Alison Hamilton, Amelia van der Merwe, Stefani du Toit, Wendy Xakayi, Xanthe Hunt

**Affiliations:** 1 Institute for Life Course Health Research, Department of Global Health, Faculty of Medicine and Health Sciences, Stellenbosch University, Bellville, South Africa; 2 Department of Psychiatry and Biobehavioral Sciences, Faculty of Health Sciences, University of California Los Angeles, Los Angeles, CA, United States of America; 3 VA Center for the Study of Healthcare Innovation Implementation and Policy, VA Greater Los Angeles Health Care System, Los Angeles, CA, United States of America

**Keywords:** acquired disability, intervention, lived experience, rehabilitation, sexual and reproductive health, women’s health

## Abstract

**Background:**

Acquiring a physical disability in adulthood necessitates a range of adjustments, with past research suggesting that some challenges encountered are unique to women. Moreover, several factors may complicate adjustment to an altered embodiment and difficulties in functioning after an accident, including insufficient rehabilitation and support services and problematic societal attitudes towards disability. In addition, women with disabilities are often excluded from health and social policy and programme development, an oversight that can result in support gaps.

**Objectives:**

This article presents the self-identified priority interventions of women with road accident-acquired physical disabilities in South Africa.

**Methods:**

We conducted interviews with 18 women with road accident-acquired physical disabilities. The participants were recruited via snowball sampling. Interviews were conducted by experienced interviewers, who were home language speakers of the participants’ preferred language of communication. The interview recordings were transcribed, translated, and coded by trained, independent researchers.

**Results:**

Study participants identified three key areas of intervention requiring consideration in supportive intervention planning: the acute post-injury environment and healthcare infrastructure, transitional services and social inclusion interventions. These were identified as overlooked areas in which they required support to successfully adapt to limitations in functioning.

**Conclusion:**

To develop inclusive, accessible, and practical policy and programming for people with disabilities, exercises like those outlined in this research – eliciting intervention ideas from lived experience – should be conducted as they highlight actionable priorities for programming.

## Introduction

### Road-traffic accidents and acquired disability in the South African context

Several 1000 people worldwide report significant injuries every day, some resulting in permanent disabilities (Krug & Sharma 2000). Road traffic injuries are the number one cause of injury-related disability (World Health Organization 2013), and the WHO estimates that 20–50 million people are injured by road-traffic accidents each year (WHO 2018). In South Africa, there are 25.1 road-accident-related deaths per 100 000 citizens reported annually (Norman et al. 2007).

There is little literature on non-fatal injuries in the country; however, extrapolation of global data suggests that South Africa’s high road-traffic accident rate would imply a similar non-fatal injury and long-term impairment rate. This is supported by road safety campaign estimates, which propose that 20 South Africans per day are involved in road-traffic accidents that leave them with a permanent impairment (Arrive Alive 2011). The prevalence rate of self-reported disability is 7.5% in South Africa, of which 2.5% of them report physical disability (Lehohla 2011). Women (8.3%) are more likely to acquire a disability than men (6.5%) in South Africa (Lehohla 2011).

Acquiring a physical disability impacts numerous spheres of life, including employment opportunities (Green et al. 2005), health care (Browne & Russell 2005), mental health (Papadakaki et al. 2017), societal participation (West, Luck & Capps 2007), and personal relationships and sexual experience (Hunt et al. 2018a; Howland & Rintala 2001; Tellier & Calleja 2017). Acquiring a physical disability also creates barriers to accessing services, spaces and opportunities (Sherry 2015). These barriers are both structural (inaccessible healthcare offices) and social (internalised prejudices held by healthcare providers) (Hunt 2018a; Lee & Fenge 2016; O’Dea, Shuttleworth & Wedgwood 2012; Pebdani, Johnson & Amtmann 2014). In addition to their individual effects, the barriers influence each other, compounding the overall effect of exclusion experienced by people with physical disabilities in South Africa (Maart & Jelsma 2014; Vergunst et al. 2015).

In the South African context, the lack of accommodating services and systems places limitations on participation and reduces opportunities for meaningful inclusion (Sherry 2015).

### Rehabilitation interventions for people with acquired disabilities in South Africa

Historically, South African disability-focussed rehabilitation has operated in an institutional context and followed an approach rooted in a medical model (Mji et al. 2013). In recent years, there have been efforts to move rehabilitation for people with disabilities from tertiary-level facility-based medical initiatives to ‘community-based rehabilitation’ (CBR) (Sherry 2015). This encompasses not only the physical service of rehabilitation but also support for people with disabilities to participate actively in society (Sherry 2015). Yet, there are gaps between how CBR is envisaged, and the reality of what is delivered to people with disabilities in South African communities, leaving the majority of the population of people with disabilities without access to adequate rehabilitation or community support services (Hanass-Hancock et al. 2017; Sherry 2015).

Even though rehabilitation, and services for people with disabilities more generally, are necessary to access education, healthcare and employment opportunities and to facilitate equitable participation in society, they are not only neglected in budget allocations but there is also a lack of human resources to deliver them (Sherry 2015).

Significant delays in the planning and implementation of rehabilitation services remain (Sherry 2015). In 2013, a task team was formed to revamp rehabilitation strategies in line with the re-engineering of primary health care (Sherry 2015). Despite the involvement of experts and successful proposals, the combination of lack of resources and synthesis with other healthcare developments impacted implementation (Sherry 2015).

Aside from rehabilitation services – with its patchy implementation and low coverage – in South Africa, the main form of intervention for people with disabilities are social protection initiatives (Sherry 2015; Zuurmond et al. 2019). These include tax rebates, subsidies for housing and targeted grants (such as the Disability and Care Dependency [DCD] grant). The DCD grant is meant to compensate for income lost because of disability-related inability to work and to cover some of the costs associated with having a disability (Kidd et al. 2018; Zuurmond et al. 2019). Whilst the grant provides some financial cushioning, it offers no assistance with other issues faced by persons with disabilities (Hanass-Hancock et al. 2017). Although provisions are made to provide housing for people with disabilities, necessary home adaptations, such as wider doors, ramps and accessible toilets and kitchens are not covered (Hanass-Hancock et al. 2017). Currently, there are only a few government programmes in South Africa focussed on addressing common difficulties experienced by people with disabilities outside of economic vulnerability.

Overall, an ever-present paradox in the healthcare system remains, particularly in low- and middle-income countries, that despite elevated health risks, people with disabilities receive consistently less care (Sherry 2015). Despite the adoption of progressive disability policy, the coverage, quality and needs-responsiveness of interventions is limited.

At the same time as services may be limited and access to those which do exist is low, the content of programmes may also not necessarily reflect service users’ priorities. Historically, services – particularly those in the medical sector – were exclusively designed, delivered and evaluated by ‘experts’, the latter being narrowly defined as various categories of medical and other professionals. However, in past decades, there has been a growing recognition of the need to involve service users – experts with lived experience – in efforts to design, improve or re-design services (Bate & Robert 2006; Bradshaw 2008; Carman et al. 2013; Crawford et al. 2002; Johnson et al. 2008). And, evidence suggests, these efforts are fruitful, with a 2018 systematic review by Bombard and colleagues of studies about engaging patients to improve services found that engaging service users could improve outcomes at the institutional and quality of care levels and was positively experienced by service users themselves.

Whilst this recognition has emerged in health services research, it certainly has precedent in the Disability Rights Movement, the clarion call of which has been ‘Nothing About Us, Without Us’. This saying highlights the importance of including experts with lived experiences in every facet of planning, policymaking, programming and evaluation, which concerns them. This involvement can be enacted through the meaningful inclusion of people with disabilities in the design and delivery of programmes (Qureshi 2020). However, whilst the ethos of ‘Nothing About Us, Without Us’ is foundational in the advocacy community, research suggests that stigma and misperceptions about the competency of people with disabilities to contribute meaningfully to programme design is a barrier to the inclusion of people with lived experience in intervention design (Qureshi 2020). As Qureshi (2020) observed, the majority of disability programming is still controlled by people who do not have a disability, and this may limit the degree to which such programming is representative of people with disabilities’ priorities (Qureshi 2020).

Considering (1) the high rates of road traffic accidents and acquired disability in South Africa, (2) the implications of acquiring a disability for women’s functioning, (3) the scarce resources available to fund supportive services, (4) the importance of centring service users’ voices in programme planning and (5) the historical exclusion of people with disabilities voices from intervention conceptualisation, this research explores South African women with acquired physical disabilities’ priorities for programming.

## Methods

The present article deals with findings from a subset of data drawn from a broader study on the sexual and reproductive health and relationship experiences of women with road accident-acquired physical disabilities. Despite the broader study’s specific focus, the data analysed for this article cover responses to more general questions about healthcare and rehabilitation, including an exploration of women’s self-identified priorities for supportive interventions after an accident.

### Study design

We conducted a cross-sectional qualitative interview study with women with acquired disabilities. This design was chosen given the research team’s interest in developing an in-depth, nuanced understanding of women’s experiences of acquiring a physical disability and its impact on their lived experience across a range of domains, as well as their self-identified priorities for post-injury support and other interventions.

### Setting

The study was conducted in Khayelitsha, a peri-urban settlement outside of Cape Town in South Africa. According to governmental records, Khayelitsha is home to 442 721 people, but unofficial estimates situate the total population at closer to two million (Sikhula Sonke n.d.; Western Cape Government 2020). This discrepancy is in part driven by the large proportion of Khayelitsha’s populace living in informal housing, or ‘shacks’, which make estimates of population size complicated. Khayelitsha has one main hospital, three provincial government clinics and a number of small municipal clinics, some of which focus on service provision for specific subpopulations, such as men or youth.

### Study population and sampling strategy

In order to establish the participant sample, snowball sampling was used. This was accomplished by distributing flyers at a research centre located in the target community of the study. The advertisement was distributed to women with physical disabilities through the knowledge networks of research assistants at the centre. The inclusion criteria for participants were that they had an acquired physical disability resulting from a motor vehicle accident,^1^ were 18 years or older, identified as a woman (both cis- and transgender women were eligible), held residence in the Western Cape, and were able to give informed consent. Each potential participant was screened, via a phone call, in accordance with the inclusion criteria. Those who met the criteria were then invited to participate and had further study information shared with them. In addition, the participants were requested to allow an hour and a half for the interview. A total of 19 women were recruited, of whom 18 went on to be interviewed.^2^ The age range of the participants was between 21 and 76 years.

### Data collection

The interviewers were selected from the research unit with which one of the lead researchers was affiliated. All interviewers included in the research process received training in disability, women’s health and principles of qualitative interviewing. The majority of the data collectors were experienced interviewers, and all were home language speakers of isiXhosa – the language of preference of the participants assigned to them.

Before the interview, a sheet detailing the study information was re-shared. After the participant had given informed consent, the interview was conducted in the participant’s home language. Each interview was audio-recorded.

After completing the interview, each participant was given a voucher to a local grocery store chain as compensation for the time and effort given to the study.

### Materials

The interview guide outlined the questions participants were asked during their interviews. The questions generally covered participants’ experience of romantic relationships, sexual and reproductive health services, and their access to these services. The focus of this article is on self-identified priorities for healthcare intervention following an acquired physical disability. The question prompts used in the interview guide included the following:

1. If you were a doctor and you wanted to set up a clinic to serve women with acquired disabilities, what would you focus on? What would you do to make your clinic and services useful to women with acquired physical disabilities?
2. If you could design a programme for women who had just been in a road traffic accident and acquired a disability, what would your programme do?

The guide was translated into isiXhosa for use by interviewers.

### Data analysis

Thematic analysis was used to analyse the data (Braun & Clarke 2006). This method was chosen for its ability to be applied to multiple data types ultimately resulting in a well-developed understanding of the reported narratives. Each transcription was translated from isiXhosa into English by a translation professional, and then back-translated and checked. Transcripts carried no identifying information on participants, but rather were marked with a participant identifier (PID), which allowed for data units (such as sentences or stories) to be connected to a single speaker.

The analysis proceeded in two stages: firstly, two independent researchers read all of the transcripts to familiarise themselves with the data set. Then, the translated interviews were coded by two independent researchers – one English speaker and another isiXhosa speaker. Each researcher read the transcripts a second time, working to identifying important units of meaning (codes). These were flagged in the transcripts using the comment function in MS Word. Once the transcripts were coded, the researchers and the study lead discussed the codes. Where there were disagreements between coders about the meaning of a specific section of the data, the isiXhosa speaker reverted to the original isiXhosa transcript and discussed the quoted text in question with the English coder and another isiXhosa speaking research team member to allow the team to arrive at an accurate interpretation and coding of the text. Based on the discussion of the codes, a preliminary sense of the data set’s main themes emerged amongst the research team. The lead author, who was responsible for the present article’s topic (self-identified priority interventions), then organised the codes into themes and developed an initial document detailing the rationale for attaching codes to specific sections of transcript, and the relationship between themes and codes.

The inclusion of a native isiXhosa speaker in the analysis was particularly valuable as isiXhosa is a richly figurative language. The meaning behind some isiXhosa figurative language can be lost in translation and thus not be coded. The researcher consulted with the data collectors and the transcribers in order to expand on the meaning included in the transcriptions. This practice of ‘cultural brokering’ can help to ensure that the participants’ experiences and contexts are correctly relayed and that meanings are preserved (Gustafsson, Norström & Fioretos 2013).

Furthermore, it is important to engage oversight researchers and independent research assistants when conducting qualitative research. This is because the potential for impacted reliability is elevated if the interpretation of multiple coders is used in analysis, as emphasised by Braun and Clarke (2006).

As outlined by Lincoln and Guba (1985), qualitative data can only be considered valuable if its trustworthiness can be confirmed. In order to maintain credibility of the data, the study utilised triangulation. As observed, both the translation and analysis processes involved a continuous feedback and revision process between multiple researchers. As a result of the nuanced nature of qualitative data, it is difficult to prove dependability and transferability. All research procedures were recorded, and demographic details about participants and the context of their experiences noted down to improve dependability of the findings and conclusions drawn and establish transferability of the study conclusions.

### Ethical considerations

A written informed consent was obtained from each participant, and the voluntary nature of the study was clearly explained. Trained research staff experienced in qualitative interviewing conducted the informed consent process. All consent and information forms were given to the participants to read or read aloud in English, Afrikaans or isiXhosa. All participants were told that they have the right to decline to participate and could withdraw from the study any time without any adverse consequences. All results were kept confidential, except where participants disclosed significant harm to themselves or others, or requested help. The risks to participants included fatigue or emotional distress when asked about sexual violence or physical trauma. Interviewers addressed these risks by reminding participants of the voluntary nature of participation and regularly checking whether they were happy to continue with the interview. In the case of emotional distress, a clear referral protocol was in place. Ethical approval for this study was obtained from the Stellenbosch University Health Research Ethics Committee (HREC) (Approval #: N19/03/037).

## Results

Amongst the issues discussed by the women, three major themes – corresponding to three different types of priority interventions – were identified. Each theme contained subthemes, and the relationship between the major themes and subthemes is laid out in Table 1.

**TABLE 1:** Major themes and subthemes.

Major themes	Healthcare infrastructure	Transition interventions	Social inclusion interventions
Subthemes	Physical accessibility of hospital and clinic infrastructure	Assistive devices	Employment and skill development
-	Disability accommodations during routine contacts	Psychosocial support	Societal attitude change
-	Healthcare worker attitudes	Empowerment-focussed programming	-
-	-	General informational needs	-
-	-	Sexual education	-

### Healthcare infrastructure

The first theme identified concerned desired supports characterised by their utility in the acute period following an accident, which resulted in a permanent impairment. This theme also concerned the accessibility of healthcare facilities to which the women required ongoing access because of their impairment and related health conditions.

Physically accessible infrastructure was central to participants’ accounts, with a particular emphasis being placed on the need to enable access to healthcare services in the form of ramps. The women raised concerns about the lack of ramps interfering with their ability to access medical care on an equal basis with non-disabled people. Participants observed that ‘ramps for entrances [*are important*] because you would find that in some clinics it is not easy to get inside’ (P1) and ‘hospitals should have ramps you can get in easily’ (P18).

Women also related that at their hospital post-injury experience was marked by difficulty in accessing ablution facilities. A number of women expressed concerns surrounding scarcity or complete lack of accessible toilets in these settings. One woman stated, ‘… you would find that they only have one toilet for people with disabilities [*sic*]’(P1). She also reported that at the clinic she now visits for routine medical services, the only accessible toilet available, ‘… is always locked and it is a storeroom’ (P1). Another woman shared:

‘[*E*]verywhere I go, before I can do anything I start by checking and go to the toilet and check if the toilet is designed [*sic*] for [*me*] who is on [*a*] wheelchair.’ (P8)

Another participant observed that in most healthcare facilities ‘toilets are not friendly for people with disabilities, so I would try to make them better so that [*disabled people*] can also feel comfortable’ (P12).

A further area which participants identified as a priority domain for the provision of post-injury support was how to negotiate routine contacts with the healthcare system once they were discharged from tertiary care. Utilising primary care facilities appeared to be an area of particular challenge, as clinics were not designed with accessibility in mind. The women found that their physical impairments simultaneously necessitated them to visit healthcare facilities more frequently than people without disabilities, and yet made waiting for extended periods of time difficult and uncomfortable. For these reasons, many participants made recommendations for post-injury support, which entailed the strengthening of primary healthcare systems to be more accessible, inclusive and accommodating of people with disabilities. For instance, some women recommended that clinics could prioritise persons with disabilities in queues. One woman suggested, ‘[*people with disabilities*] are the ones that the clinics should attend to first… because the clinics we go to are packed’ (P3), whilst another observed that she ‘would like people with disabilities to be given priority than those who went to the clinic for minor things’ (P14). Participants’ desire to see long waiting times at clinics reduced and difficulties in navigating the built environment of healthcare facilities addressed, should also be seen within the broader context of difficulties in getting to healthcare facilities. Many participants find it difficult to get transport facilities for going to clinics and hospitals. This led to the identification of an additional priority for intervention, with one woman suggesting that ‘… [*it would help if*] there can be a minibus service from the clinic… then again after check-up take them back home’.

Finally, a number of participants expressed the need for more empathic and informed service from healthcare workers. Women noticed a desire for ‘[*a*] clinic that cares’ (P14), and the need to ‘make sure that [*people with disabilities*] are treated well’ in healthcare settings (P10). Women referred to experiences of poor treatment and marginalisation within the healthcare system, both during the acute post-injury phase and in routine contacts, and call for more respectful services responsive to patients with disabilities.

### Transition interventions

The process of transition (adjustment to functioning as a person with a physical disability) was another area highlighted by the participants as full of opportunities for supportive interventions. Transition interventions suggested by the participants focussed on assisting women with acquired disabilities to adjust to their new embodiment and functioning. A number of study participants reported a notable lack of support during this transition phase and made suggestions for how this could be addressed.

One common recommendation regarded the need for widespread provision of wheelchairs and wheelchair support. Women stated that ‘… mak[*ing*] sure that everybody has got a wheelchair that is in good condition’ (P8) was imperative, so that a lack of access to this assistive technology would not be a barrier to their participation.

Many of the participants in the study also stressed the need for support beyond assistive devices, most notably in the form of psychosocial support. Some of the women suggested support groups, and:

[*H*]av[*ing*] a centre whereby [*women with physical disabilities*] can meet all [*together*] to discuss things that concern such women.’ (P1)

Another participant shared the desire to spend time with other women who could mirror her own experience and with whom she shared a common ground. She said, ‘I wish sometimes that [*I can spend time with*] someone [*who*] feels what I feel… not to just come and talk about something you don’t know’ (P2). Other women suggested that the introduction of support groups for women with physical disabilities in their communities would allow women to access needed information, for instance, how to do housework after acquiring a disability: ‘… as women that are living with disabilities [*I would*] like [*to*] talk about how [*I could*] help in house chores’ (P1).

Several respondents highlighted the need for empowerment-focussed programming, explaining:

‘I wish that [*women with acquired physical disabilities*] can learn to be independent not to depend [*on*] a partner you see. [*I*] wish that they can learn to be independent, if you have children just look after your children and be independent without depending on someone for your life to move on.’ (P2)

Another woman noticed the need for programmes focussed on building the self-esteem of women with acquired disabilities:

‘[*A programme*] would encourage the women with disability… You see it hurts to be undermined especially when you were born normal [*sic*] and see yourself people with disabilities it’s not easy to accept even for me – you see that crutch – I couldn’t use it, you see when I see people passing by me I would struggle and end up falling down, when you are walking with other people you forgot that crutch because you are not used to it you used to walk for yourself it’s not easy.’ (P3)

The desire to impart encouragement and support the development of a positive identity and self-esteem as a women with an acquired disability was echoed by others, with one woman opining: ‘I would like to add by telling [women with acquired physical disabilities] that they need to be confident about their bodies’ (P8).

Another woman stated the desire for a more general type of support group here peers could share information. She said:

‘I [*would suggest*] a programme that gather[*s*] you together as women and [*allows you to*] advise each other that no when life is like this, this is what you need to do because some of us are still married or others got married after the disability. So that is the way that you can relate even with those who [*have*] not yet accepted [*that they have difficulties in functioning*] and all that, so that is what I can do.’ (P18)

This need for information was seconded by another participant who observed the need for programming, which ‘teaches one to seek knowledge about being disabled’ (P6).

A similar desire for improved knowledge underlay several participants who expressed the need for sexual education for women with physical disabilities. Participants noticed that such programmes could not only serve an informational role, informing women with physical disabilities about matters of sexual health but also promote women’s autonomy in sexual relationships. As one participant stated, ‘I would teach them when they visited my clinic, teach them about sex’ (P12).

### Social inclusion interventions

There was a final cluster of suggested interventions, which were responses to the need for broader changes to be made to the participants’ economic, social and personal environment in order to facilitate their adjustment, functioning and participation.

Participants expressed need for employment and skill development for women with acquired physical disabilities. The main motivation for this request was economic security. One woman said:

‘I would try something like farming. So that people can have jobs and not rely on grant money. It must be owned by us, we must not have people who are not disabled helping us.’ (P17)

Furthermore, women emphasised the need for reasonable accommodation in employment. One of the participants said, ‘I was supposed to work at Civil Centre and at Mitchell’s Plain but there are stairs. So I couldn’t’ (P16).

Others echoed this sentiment regarding the need for economic opportunities to facilitate independence and – centrally – to allow women to continue to fulfil their roles as providers for their children. One woman explained:

‘[*We need*] things such as jobs, whether a person is doing a hand work or sewing but something that is going to keep her busy, so that she can also see that she is important not important by getting grant money no, but something that can also help her in future be able to work for her children like everyone else.’ (P16)

Another respondent called for ‘something that will make us money … so that those who have children can be able to take care of their children’ (P17).

Finally, participants highlighted the need for attitude change around disability. ‘Teach people that people with *disabilities* are also humans, they can also do things that people without disability can do’ (P8), one woman explained, whilst another suggested the need for education, which would change the stigmatising behaviour of people without disabilities towards people with disabilities in the community:

‘The first thing is that when you see a disabled person, don’t be like you are seeing an animal or a strange thing. Look at that person and put her closer to you, after that check what her problem is, sit down with her and ask her questions although you are also disabled. Talk to that person because it might happen that as you are disabled also but maybe she is disabled more than you, understand.’ (P9)

## Discussion

The purpose of this research study was to identify priorities of South African women with physical disabilities for intervention after acquiring a physical impairment. Firstly, the theme identified in this study concerned the quality and accessibility of health care. Participants emphasised the need for ramps and accessible ablution facilities at healthcare facilities, shorter waiting times at clinics, and more informed and empathic treatment by healthcare professionals. Secondly, participants identified challenges encountered whilst adjusting to an altered embodiment and difficulties in functioning. Within this theme women emphasised the importance of assistive devices such as wheelchairs, and psychosocial support, as potential enablers of adjustment. In addition, participants pointed to the need for sexual and reproductive healthcare services and education to be strengthened. Thirdly, women highlighted the need for broader changes to their environments to facilitate social inclusion, particularly the provision of economic opportunities, employment and skill development to enhance their independence.

Our findings echo a number of past qualitative studies on the experience of individuals with disabilities, many of which contain reports of discontent with the state of disability services, including in terms of health care (Ganle et al. 2016; Rugoho & Maphosa 2017; WHO 2011; Zuurmond et al. 2019). The need for accessible rehabilitation and health services, including assistive devices, is well documented (Maart & Jelsma 2014). Individuals with physical disabilities have a significantly higher likelihood of having unmet healthcare needs, and like our participants, being female, experiencing poverty and being unable to access private health care, contribute to this risk (Mahmoudi & Meade 2015; Sakellariou & Rotarou 2017; Smith 2008). Equitable access to care is important as it has both individual and broader structural ramifications, which impact the person with the disability, including physical, social, psychological and economic well-being, as well as independence (Neri & Kroll 2003).

The negative experience of healthcare and rehabilitation services, general healthcare services, and exposure to lack of knowledge and negative attitudes amongst healthcare providers found in this study have been reported elsewhere (Maart & Jelsma 2014; Sakellariou & Rotarou 2017). Previous research studies support our findings that people with physical disabilities need more informed and empathic care from health professionals, and the results of this enquiry point to the need for strengthened training of health professionals to work with people with disabilities (Au & Man 2006; Kirschner & Curry 2009).

It is notable that despite the present data being drawn from a study concerning sexual and reproductive health, the themes of sexual and reproductive health and sexuality did not feature prominently in women’s priorities. Indeed, the kinds of interventions or supports that women wanted had more to do with enabling access to basic services and participation in their communities, including access to work, than they did with the kinds of things which usually comprise rehabilitation programming. However, this finding is supported by past work. In Auger et al.’s (2020) study on the perceived priorities and needs regarding sexuality for individuals going through stroke rehabilitation, support for basic activities of daily living, such as eating, communicating and walking, was perceived as more important and a bigger priority than sexuality.

However, similar to our findings, Auger et al. (2020) also reported that – despite the prominence of basic needs such as access to work and health care – there was a desire to address sexuality in some form. The relative importance of sexual and reproductive health and sexuality may change over time, with its salience increasing after the acute and post-acute phases of acquiring a physical impairment. For instance, Leibowitz (2005) reported that sexuality was not a priority soon after injury for a majority of the women interviewed in their study, but that it became more important later on in the women’s lives.

A key strength of this study is the foregrounding of women’s experience of healthcare services for persons with disabilities, and the creation of a platform for the self-identification of programming in need of strengthening. A potential limitation of the study is that all of the participants reside in the same geographical area and were recruited using snowball sampling, thus limiting the transferability of the findings. In order to gain a more generalisable sense of South African women with disabilities’ intervention priorities, the same study could be repeated in other areas. However, it would likely be more impactful for the processes of this study to be taken up by national, subnational and private service providers for intervention planning and delivery.

Indeed, this study has demonstrated, in a small sample, that experts with lived experience of an acquired physical disability have clear ideas about the kinds of services and support, which would benefit them. Bombard et al. (2018) observed that when service users are involved in intervention planning and design, it results in shifts in organisational culture promoting further patient participation, opportunities for collaboration and mutual learning between healthcare professionals and service users and could result in the correcting of power imbalances between providers and service users in healthcare settings. At the very least, this study has shown the potential for meaningfully including women with acquired physical disabilities in the design of post-injury programming to identify opportunities for intervention.

## Conclusion

The study findings point to the multiple barriers faced by women with disabilities in their attempts to access health care and adjust to difficulties in functioning after acquiring a physical disability. The supporting literature in this field shows that the risk of unmet healthcare needs is exponentially increased for those who are women, poorer and have greater difficulties in functioning. These women are an underserved population and need to be meaningfully included in programme planning, design, monitoring and evaluation so that their needs and priorities are adequately met. In this manner, we are more likely to have policies and intervention strategies that are representative of the community they serve, accessible to its users, and inclusive for all.

## Footnotes

1 The main aim of this study was to explore experiences of women with acquired disabilities resulting from motor vehicle accidents. In particular, the study aimed to focus on these women’s experiences with relationships, their sexuality, and their reproductive and sexual health. This particular subgroup of the South African population was chosen because of their significance to public health and the prevalence of motor vehicle accidents in the country.

2 The woman who opted out of the study after recruitment was advised by the administrator of the financial settlement from the accident in which she had acquired a physical impairment not to participate in the study. Despite efforts made by the study lead – the last author of this article – to explain the purpose of the study to the woman and the administrator, he advised her not to take part.
